# An image quality comparison study between XVI and OBI CBCT systems

**DOI:** 10.1120/jacmp.v12i2.3435

**Published:** 2011-02-04

**Authors:** Srijit Kamath, William Song, Alexei Chvetsov, Shuichi Ozawa, Haibin Lu, Sanjiv Samant, Chihray Liu, Jonathan G. Li, Jatinder R. Palta

**Affiliations:** ^1^ Yale‐New Haven Hospital New Haven CT 06510‐3202 USA; ^2^ Department of Radiation Oncology University of California San Diego Rebecca and John Moores Comprehensive Cancer Center La Jolla CA 92093‐0843 USA; ^3^ University of Florida Proton Therapy Institute Jacksonville FL 32206 USA; ^4^ Juntendo University Grduate School of Medicine Bunkyo‐ku Tokyo 113‐8421 Japan; ^5^ Department of Radiation Oncology University of Florida, Davis Cancer Center Gainesville FL 32610‐0385 USA

**Keywords:** cone‐beam CT, image quality, XVI, OBI, image‐guided radiotherapy (IGRT)

## Abstract

The purpose of this study is to evaluate and compare image quality characteristics for two commonly used and commercially available CBCT systems: the X‐ray Volumetric Imager and the On‐Board Imager. A commonly used CATPHAN image quality phantom was used to measure various image quality parameters, namely, pixel value stability and accuracy, noise, contrast to noise ratio (CNR), high‐contrast resolution, low contrast resolution and image uniformity. For the XVI unit, we evaluated the image quality for four manufacturer‐supplied protocols as a function of mAs. For the OBI unit, we did the same for the full‐fan and half‐fan scanning modes, which were respectively used with the full bow‐tie and half bow‐tie filters. For XVI, the mean pixel values of regions of interest were found to generally decrease with increasing mAs for all protocols, while they were relatively stable with mAs for OBI. Noise was slightly lower on XVI and was seen to decrease with increasing mAs, while CNR increased with mAs for both systems. For XVI and OBI, the high‐contrast resolution was approximately limited by the pixel resolution of the reconstructed image. On OBI images, up to 6 and 5 discs of 1% and 0.5% contrast, respectively, were visible for a high mAs setting using the full‐fan mode, while none of the discs were clearly visible on the XVI images for various mAs settings when the medium resolution reconstruction was used. In conclusion, image quality parameters for XVI and OBI have been quantified and compared for clinical protocols under various mAs settings. These results need to be viewed in the context of a recent study that reported the dose‐mAs relationship for the two systems and found that OBI generally delivered higher imaging doses than XVI.^(1)^

PACS numbers: 85.57.C‐, 85.57.cj, 85.57.cm, 85.57.cf

## I. INTRODUCTION

Cone beam computed tomography (CBCT) technology has recently been integrated with the conventional linear accelerators to provide volumetric imaging at the time of treatment setup.^(^ ^1^ ^–^ ^6^ ^)^ Prior to its introduction into the clinic, patient positioning at treatment time was achieved by means of two‐dimensional projection images obtained using the mega‐voltage portal imaging devices. The advent of three‐dimensional imaging and visualization during treatment has provided new opportunities and challenges for improved patient positioning.^(^ ^7^ ^–^ ^12^ ^)^ While acquisition of daily CBCT has enabled improved visualization and localization of tumors,^(^ ^11^ ^,^ ^12^ ^)^ it comes at a cost of added patient dose,^(^ ^1^ ^,^ ^13^ ^)^ which can be a cause of concern. For example, if a dose of 2 cGy is delivered per scan during each of 40 fractions, it amounts to accumulated dose of 0.8 Gy over the course of treatment. The dosimetric properties of Elekta's XVI CBCT system were studied by Islam et al.^(14)^ Skin dose measurements for prostate patients on Varian CBCT have been reported by Wen et al.^(15)^ Since the primary purpose of the CBCT is to provide a volumetric image for patient positioning, it is important to study the dose–image quality tradeoffs for CBCT. While it is necessary for CBCT image quality to be sufficiently good for accurate patient positioning through rigid registration with the planning CT image, improved CBCT image quality could enable its accurate deformable registration with the CT.^(16)^ The deformation information could be used, then, for real‐time treatment planning or for CBCT based treatment dose computation^(^ ^16^ ^,^ ^17^ ^)^ to accumulate fractional patient doses for the purpose of adaptive therapy. Compared to the relatively narrow fan beam geometry of a conventional CT scanner, the CBCT collimates a wide cone beam. Naturally, X‐ray scatter is significantly greater in CBCT as compared to the CT^(^ ^3^ ^,^ ^18^ ^)^ and this presents challenges in obtaining images of quality acceptable for image registration. For example, scatter results in occurrence of cup and streak artifacts.^(3)^ Image artifacts in CBCT may have other causes as well. A bad pixel in the kV imaging panel may result in ring artifacts.^(19)^ Residual signals from previous acquisitions when visible on newly acquired images may result in ghost artifacts.^(20)^ Both pre‐ and postreconstruction methods have been proposed to alleviate the effects of various artifacts on CBCT image quality.^(^ ^18^ ^,^ ^21^ ^–^ ^23^ ^)^ CBCT image quality has also been a subject of some investigations.^(^ ^24^ ^–^ ^26^ ^)^ A quality assurance program for CBCT including image quality has been developed.^(27)^ Image quality data based on CBCT QA was recently reported.^(28)^ Dose and image quality data^(24)^ for a cone beam C‐arm CT system^(^ ^29^ ^,^ ^30^ ^)^ and similar data^(^ ^25^ ^,^ ^31^ ^)^ for a MV CBCT system^(^ ^32^ ^,^ ^33^ ^)^ have been published.

Two commercially available and widely used CBCT systems, Elekta's XVI and Varian's OBI, are available at our institution. Recently, Song et al.^(1)^ made extensive dose measurements on XVI and OBI systems for clinical protocols using standard dose phantoms. They measured half value layers (HVL) to characterize the beams for the two systems. They also established an expected linear relationship between mAs and dose for the two systems, and summarized the similarities and differences between the two systems. Since the primary purpose of the CBCT is to provide images suitable for accurate patient positioning through image registration, the ultimate goal of this line of research is to evaluate the impact of increasing imaging dose on the image quality. Therefore, the purpose of this work is to derive image quality vs. mAs relationship for the two commercial systems under standard clinical settings and thereby infer the desired relation between dose and image quality. Image quality parameters evaluated include high‐contrast resolution, low contrast resolution, contrast to noise ratio, image uniformity and noise.^(27)^ We believe that this work will provide valuable information about dose–image quality tradeoffs for those who have implemented or wish to optimize CBCT scanning protocols for their specific clinical applications.

## II. MATERIALS AND METHODS

### A. CBCT systems evaluated

Both systems evaluated in this study have the capability to image in either radiographic (2D), fluoroscopic (2D+time), or volumetric (3D) modes. For simplicity in comparison, we evaluated in 3D mode only. The first system evaluated was the X‐ray Volumetric Imager (XVI, Elekta Oncology Systems, version 4.0) integrated with the Elekta Synergy linear accelerator platform.

The system consists of a kV X‐ray source (70–150 kVp) with a flat‐panel, amorphous silicon (aSi)/cesium iodide (CsI) detector. The KV X‐ray source and detector are mounted on the gantry such that the axis that passes through their centers is always perpendicular to the MV collimator axis. The second system evaluated was the On‐Board Imager (OBI, Varian Medical Systems, version 1.4) integrated with the Varian Trilogy unit. This system, similar to XVI, consists of a kV X‐ray source (KVS) with a flat‐panel aSi detector (KVD) mounted orthogonal to the gantry axis using a robotic arm (Exact). (For a detailed description of the two CBCT systems and their similarities and differences, the reader is referred to Song et al.^(1)^). For XVI, four clinical protocols were used, namely, “head & neck”, “prostate”, “pelvis”, and “chest”. Since the goal is to study the effect of mAs on image quality for each protocol, four wide‐ranging mAs settings were used to acquire data. Table 1 details the machine settings used for each protocol for XVI. The kV collimators used are labeled such that the field of view (FOV) settings ‘S’ (27.68×27.68 cm FOV), ‘M’ (42.64×42.64 cm FOV) and ‘L’ (52.40×52.40 cm FOV) are used in combination with length settings ‘10’ (13.54 cm length) and ‘20’ (27.67 cm length). For OBI, the images were acquired using both the full‐fan (24×24 cm FOV) and half‐fan (45×45 cm FOV) modes that are respectively used with the full and half bow‐tie filters. In this study, we did not perform measurements for OBI without filters, since no clinically useful mode involves absence of filter. The wide‐ranging mAs settings were used for each of the FOV settings to study their effects on the image quality. Table 2 details the machine settings for each protocol on OBI. Note that the purpose of the bow‐tie filters is to improve the CBCT image quality and to reduce X‐ray scatter reaching the detector, as well as reduce dose to the patient.^(34)^ We opted to take single measurements for this study because several image quality metrics were quantified under multiple protocols each with multiple settings. The required measurements became prohibitively large, especially with X‐ray tube frequently overheating.

**Table 1:** List of the relevant parameters used in the scanning with the XVI unit. For the acquisition gantry angle, cw means clockwise rotation and ccw means counter‐clockwise rotation.

*Protocol*	Head & Neck	Prostate	Pelvis	*Chest*
kV Collimator	S20	M10	M20	L20
kVp	100	120	120	120
(mA,ms/frame)	(10,10), (10,20), (25,10), (20,20)	(25,20), (25,40), (40,32), (50,40)	(25,20), (25,40), (40,32), (40,40)	(25,20), (25,40), (40,32), (50,40)
# frames	361	650	650	650
Total mAs	36.1, 72.2, 90.25, 144.4	325, 650, 832, 1300	325, 650, 832, 1040	325, 650, 832, 1300
Acquisition Gantry Angle	260° to 100° *cw*	183° to 179° *cw*	183° to 179° *cw*	183° to 179° *cw*
Acquisition Time	~ 70 sec	~ 120 sec	~ 120 sec	~ 120 sec
Voxel Size	1×1×1 mm	1×1×1 mm	1×1×1 mm	1×1×1 mm

**Table 2:** List of the relevant parameters used in the scanning with the OBI. For the acquisition gantry angle, cw means clockwise rotation and ccw means counter‐clockwise rotation.

*Protocol*	Full‐fan	*Half‐fan*
Filter	Full Bow‐tie	Half Bow‐tie
kVp	125	125
(mA, ms/frame)	(10,10), (25,20), (50,20), (50,30), (80,25), (80,32)	(10,10), (25,20), (50,20), (50,30), (80,25), (80,32)
# frames	630	630
Total mAs	63, 315, 630, 945, 1260, 1612.8	63, 315, 630, 945, 1260, 1612.8
Acquisition Gantry Angle	175.5° to 182.5° *cw* or 184.5° to 177.5° *ccw*	175.5° to 182.5° *cw* Or 184.5° to 177.5° *ccw*
Acquisition Time	~ 60 sec	~ 60 sec
Voxel Size	0.47×0.47×2.5 mm	0.88×0.88×2.5 mm

### B. Implementation

All scans were saved and exported as DICOM files. The DICOM files were read using the DICOM reader in MATLAB (The MathWorks Inc., Natick, MA). MATLAB code was used to manipulate the images. Graphical User Interface (GUI) was developed to scroll through the slices of an image, select regions of interest (ROI) on slices, and obtain mean and standard deviations of pixel intensities over the area defined by ROIs. The GUI was used to select ROIs on fixed locations on the same slices of each of the CATPHAN images and to evaluate the various image quality indices.

In general, the pixel values read from a DICOM image file are not their CT number values. Rather:

(1) CT number=pix×RescaleSlope+RescaleIntercept

where *pix* is the pixel value for each pixel. RescaleSlope and RescaleIntercept values can be obtained from the metadata in the DICOM image file and may vary from system to system. The RescaleSlope value is observed to be a unity for both OBI and XVI images. The RescaleIntercept values are found to be −1000 and −510, respectively, for OBI and XVI images. The XVI scanner was not calibrated for CT numbers and so application of the above formula does not necessarily yield the correct CT number. For the OBI, a calibration was performed during installation, and it was verified during acceptance testing that the CT number for air, acrylic and LDPE were within 40 units of their nominal values. In any case, we wish to clarify that we have used the pixel values to report some results that follow and the numbers may, therefore, be quite different from the CT number of the imaged object, especially for XVI. Note that since RescaleSlope=1 for both systems, differences in pixel values of two regions should equal the CT number (Hounsefield unit) difference between the two regions.

### C. CATPHAN phantom

**Figure 1:**
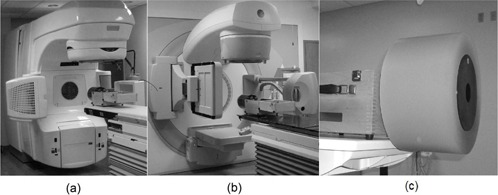
The two major commercial CBCT systems evaluated: (a) the OBI, (b) the XVI, with their corresponding CATPHAN phantom (small phantom) setup shown, and (c) the CATPHAN phantom with the annulus surrounding it (large phantom).

The CATPHAN 504 phantom (model: CTP 504, The Phantom Laboratory) was used to evaluate CBCT image quality. The phantom is composed of several modules that can be used to measure various image quality indices. (For details of the CATPHAN phantom, the reader is referred to the CATPHAN manual: CATPHAN 500 and 600 manual, The Phantom Laboratory, available from http://www.phantomlab.com)). Note that the phantom has a diameter of 20 cm (head and neck size). It was used to evaluate all four protocols for XVI and both scanning modes (half‐fan and full‐fan) for OBI. Additionally, a uniformity body annulus of 32 cm diameter that slides and fits around the CATPHAN was used to mimic body imaging attenuation for prostate, pelvis and chest protocols for XVI and half‐fan mode for OBI. The phantom (with or without the annulus) is positioned off the edge of the treatment couch by mounting and suspending it from the case supplied with the phantom. We will refer to the phantoms with and with out the annulus, respectively, as the large phantom (LP) and small phantom (SP). (Figures 1a)and (1b) depict the two CBCT systems studied with the small phantom positioned on the couch. (Figure 1c) depicts the large phantom. Radio‐opaque markers (BBs) on the exterior of the phantom are aligned with the laser and used to maintain consistency in positioning the phantom. The tests performed and the modules involved are described below.

**Figure 2:**
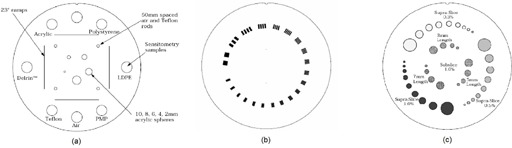
Three of the modules of the CATPHAN phantom (model: CTP 504) and the image quality parameters we have measured using them: (a) the CTP 404 module for pixel value stability and contrast‐to‐noise ratio, (b) the CTP 528 module for high contrast resolution test, and (c) the CTP 515 module for low contrast resolution test.

**1. Pixel value stability (with varying mAs) and contrast‐to‐noise ratio (CNR):** CTP 404 (Fig. 2(a) consists of cylindrical targets of materials of seven different known densities, viz., air, polystyrene, LDPE, PMP, Teflon, derlin and acrylic (Table 3). The mean and standard deviations of CT numbers in each insert are computed and tabulated. Ideally, the mean observed CT number should be within 40 units of the nominal CT number (as per the CATPHAN manual) of each insert provided by the manufacturer and should be independent of the scanning protocol. Standard deviation of pixel values in any region of uniform density is indicative of noise in that region the image. CNR was calculated as

(2) CNR=(S (ROI)−S (BG))/σ

where *S (ROI)* and *S(BG)* are the mean pixel values over a ROI in an insert and in the background, and σ is the standard deviation of the pixel values in a region of interest in the background. Specifically, the polystyrene insert was used for the CNR computation as it has the lowest contrast with the background (see Table 3). The inserts have a diameter of about 1.2 cm. Circular ROIs of 0.6 cm diameter were used so as to be completely contained within the inserts.

**Table 3:** Materials present in CTP 404 and their nominal CT numbers.

*Material*	*Nominal CT #*
Air	−1000
PMP	−200
LDPE	−100
Water	0
Polystyrene	−35
Acrylic	120
Derlin	340
Teflon	990

**2. Noise and uniformity:** CTP 486 is a uniform density module such that the CT number of the module is within 2% of the CT number of water. Standard deviation of pixel intensities over a region of interest (ROI) is indicative of the image noise. We used circular ROIs of 0.6 cm diameter for evaluating noise. Because of the presence of a “cupping” effect and the fact that it led to an increased standard deviation with ROI size, we did not use larger ROIs to evaluate noise. We report the mean of the noise evaluated in five ROIs that are located at the center and four peripheral regions of the phantom. Stability of pixel values from the periphery to the center of an image slice of a uniform density material is an indication of image uniformity. If there is much variation of pixel values in regions at the periphery from those at the center, the image is considered to be nonuniform. Therefore, image uniformity is measured using:

(3) Uniformity index (UI)=S (Peripheral‐ROI)−S (Center‐ROI)

where *S (Peripheral‐ROI)* and *S (Center‐ROI)* are the mean CT numbers in ROIs at the periphery and center, respectively, as defined by Stützel et al.^(26)^ Therefore, if the UI is positive, this would indicate a “cupping” artifact; if the UI is negative, then it would indicate a “capping” artifact, the degree of which will depend on the absolute value of the corresponding UI.

**3. High‐contrast (spatial) resolution:** High‐contrast resolution is the property of being able to distinguish between two closely spaced objects in an image. It can be measured by examining how closely spaced lines can be resolved in an image generated by the scanning system. The CTP 528 module (Fig. 2(b) consists of 1 through 21 line pairs per centimeter (lp/cm) to measure the high‐contrast resolution. High‐contrast resolution is measured in lp/cm and is a visual test; therefore, this test is somewhat subjective in nature.

**4. Low‐contrast resolution:** Low‐contrast resolution is the property of an image of being able to distinguish between various contrast materials. The low‐contrast module, CTP 515 (Fig. 2(c) consists of supra‐slice and sub‐slice targets. The supra‐slice targets are of three contrast levels: .3%, .5% and 1%. There are nine supra‐slice targets of each contrast level with diameters 2, 3, 4, 5, 6, 7, 8, 9 and 15 mm, respectively. The number of supra‐slice targets visible on a scan is indicative of the low contrast resolution. The sub‐slice targets vary in length in the direction perpendicular to the image slice plane and their visibility is a function of the slice resolution. Therefore we opted to omit the sub‐slice contrasts from our evaluation.

Based on central and peripheral dose measurements using cylindrical ‘head’ (18 cm diameter) and ‘body’ (30 cm diameter) dose phantoms, Song et al.^(1)^ established a linear relationship between mAs and dose, for each of the scanning protocols for both CBCT systems. They observed that OBI generally delivers higher doses than XVI for scans with the same mAs. From this general fact, it would be expected that OBI would generally exhibit better image quality than its counterpart XVI, provided detector noise and scatter geometry are similar. It is to be noted that the CATPHAN phantom used in this study is an image quality phantom and, hence, one cannot directly infer the dose received for a given image quality using the image quality vs. mAs relationship derived in this work together with the work of Song et al.^(1)^ However, an approximate estimate of the imaging dose can thus be obtained.

## III. RESULTS

### A. Pixel value stability

**Figure 3:**
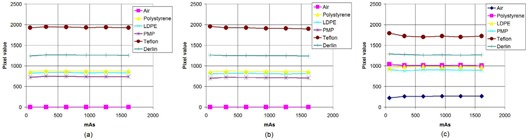
Mean pixel values in ROIs of various inserts of the CTP 404 module as the mAs is varied for the OBI scanning protocols: (a) full‐fan (small phantom (SP)), (b) half‐fan (small phantom (SP)) and (c) half‐fan (large phantom (LP)).

**Figure 4:**
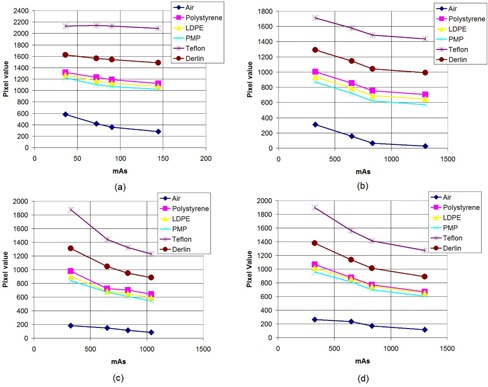
Mean pixel values in ROIs of various inserts of the CTP 404 module as the mAs is varied for the four XVI scanning protocols using the small phantom (SP): (a) head and neck, (b) prostate, (c) pelvis, and (d) chest.

**Figure 5:**
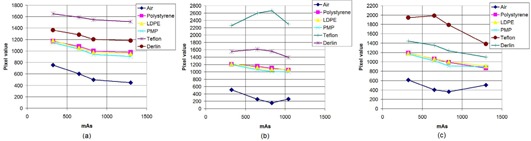
Mean pixel values in ROIs of various inserts of the CTP 404 module as the mAs is varied for three of the XVI scanning protocols using the large phantom (LP): (a) prostate, (b) pelvis, and (c) chest.

The average pixel values for circular 0.6 cm diameter ROIs in various inserts of CTP 404 for OBI are reported in Fig. 3. It is observed that the pixel values of an insert are quite stable over the range of mAs settings for a fixed scanning mode. The variations are generally within a 40 HU range (since pixel value difference equals HU difference for these systems, as previously stated)for all inserts over the entire mAs variation for both the full‐fan as well as half‐fan scans with the exception of an outlier or two. The CT numbers calculated as explained previously from the pixel values are found to be shifted by an amount varying approximately between −100 HU to 100 HU from the expected CT numbers (as per the CATPHAN manual) depending on the insert, the scanning mode (half‐fan/full‐fan) and the phantom size. For example, the correct CT number of PMP is −200 HU, while that computed from the pixel values in the half‐fan images of the large phantom turns out to be in the vicinity of −100 HU. For XVI, the average pixel values (hence CT numbers) in circular ROIs within inserts of CTP 404 are found to be a function of mAs. For the small phantom, for each of the protocols – viz., head and neck (full‐fan, Fig. 4(a), prostate (half‐fan, Fig. 4(b), pelvis (half‐fan, Fig. 4(c) and chest (half‐fan, Fig. 4(d) – it is found that the pixel value decreases with increasing mAs. The range in pixel value variations for most inserts is found to be over 300 HU over the mAs range used for each protocol. The same variation is also observed in case of prostate protocol (half‐fan, Fig. 5(a) when the large phantom is used. For pelvis (half‐fan, Fig. 5(b) and chest (half‐fan, Fig. 5(c) protocols, a different behavior is observed when the large phantom is used. For higher density inserts (e.g., Teflon), the pixel value initially increases and then decreases with increasing mAs. For intermediate density inserts (e.g., PMP and LDPE), pixel value monotonically decreases with increasing mAs. For low density inserts (example: air), pixel value first decreases and then increases with increasing mAs when scanning using these protocols. Due to the variation of pixel values with mAs, it is to be noted that the CT numbers so calculated from the DICOM information of the scans for XVI may not be used to estimate the actual CT numbers if suitable corrections are not applied.

### B. Noise

**Figure 6:**
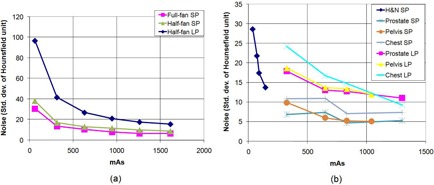
Noise averaged over the five ROIs chosen in the CTP 486 module as the mAs is varied, using the small phantom (SP) and large phantom (LP), for (a) OBI and (b) XVI.

Figure 6 shows the mean value of the noise at five ROIs of CTP 486. For OBI, as expected, the noise is observed to asymptotically decrease with increasing mAs (Fig. 6(a). An overall reduction in the noise with mAs is also observed for XVI (Fig. 6(b). The head and neck protocol is set at low mAs settings (36.1 to 144.4) and is used only with the small phantom, while the three remaining protocols are set with higher mAs (325 to 1300) and are used with small and large phantoms. A steady decrease in noise with increasing mAs is seen for the head and neck protocol. The magnitude of noise somewhat stabilizes with small fluctuations at higher mAs for the other protocols.

### C. Contrast‐to‐noise ratio (CNR)

**Figure 7:**
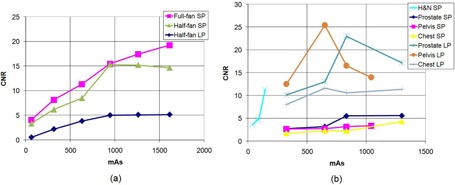
Contrast‐to‐noise ratio measured using the polystyrene ROI and the background material (water‐equivalent) of the CTP 486 module as the mAs is varied using the small phantom (SP) and large phantom (LP), for (a) OBI and (b) XVI.

The CNR generally increases with increasing mAs (Fig. 7(a) for OBI. This is because noise decreases with increasing mAs and pixel values are relatively constant with varying mAs. For the half‐fan scans, the CNR saturates at higher mAs where beyond about 1200 mAs, there is not additional benefit. This, perhaps, is the reason why the OBI scan protocols used in the clinic (i.e., half‐fan and full‐fan) are set to a default value of 1260 mAs. For XVI, the increase in CNR with mAs (Fig. 7(b) is systematic at low mAs (head and neck) and marginal and variable for higher mAs (prostate, pelvis and chest), especially for the small phantom. This behavior can be explained from the fact that for the small phantom, the noise slightly fluctuates with mAs for higher mAs values while the pixel value of ROI decreases with increasing mAs for the polystyrene insert, which is used for CNR computation.

### D. Uniformity

**Figure 8:**
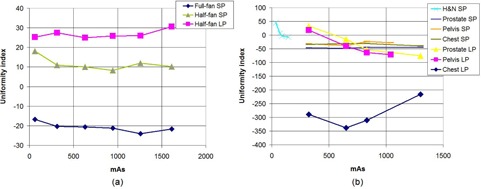
Image uniformity index (UI) measured on the CTP 486 module as the mAs is varied, using the small phantom (SP) and large phantom (LP), for (a) OBI and (b) XVI. Positive value indicates a ‘cupping’ artifact, whereas a negative value indicates a ‘capping’ artifact.

The uniformity index was measured using the ROIs at the center and periphery of the uniform phantom. It has an average value of −20.78 for full‐fan scan and averages of 11.58 and 26.72 for half‐fan scan with the small and large phantoms, respectively, for OBI (Fig. 8(a). (Figure 8b) shows the values of the uniformity index for various scanning protocols and mAs settings for XVI. The average values of the uniformity index is 10.38 for the head and neck protocol (small phantom only) and −46.12 (−27.70), −30.41 (−39.39) and −33.47 (−288.67) for prostate, pelvis and chest protocols with the small (large phantoms).

### E. Low‐ and high‐contrast resolutions

The visibility of the low‐contrast targets for OBI improves with the increase in mAs, using the small phantom. While none of the targets were visible for the lowest mAs setting (i.e., 63 mAs), up to 6 and 5 discs, respectively, of 1% and 0.5% contrast were visible for the highest mAs setting (i.e., 1612.8 mAs) using the full‐fan mode with the small phantom, and up to 6 and 1 of the discs were visible for the half‐fan mode with the small phantom at the same mAs setting. However, none of the low‐contrast targets are visible on OBI images for the half‐fan mode with the large phantom. Also, for the low‐contrast resolution test, none of the low‐contrast targets are visible in the XVI images in the mAs range tested (i.e., up to 1300 mAs for the chest protocol) for either phantom.

For the high‐contrast resolution test, the OBI full‐fan mode exhibits consistency with about 10 lp/cm visible for all mAs settings for both phantoms. For half‐fan scan, 6 lp/cm are visible for all mAs and does not exhibit improvement as a function of mAs, either. For the XVI, 4 lp/cm are consistently visible for all mAs settings tested for all protocols on both phantoms, also without exhibiting improvement with the mAs when the medium resolution reconstruction quality is used, as explained in the following discussion section.

## IV. DISCUSSION

The purpose of this work is to compare the image quality of two popular commercial systems, specifically, to study the influence of mAs (and hence dose) on image quality parameters. We wish to emphasize that, while the quality of individual components of the imaging system (the X‐ray generator subsystem, the flat‐panel detector subsystem and the image reconstruction and enhancement software subsystem) has a bearing on the image quality, our goal is to study the quality of images generated by the entire CBCT system under typical clinical settings, and its dependence on mAs.

Before discussing the results, we review some factors that one needs to keep in mind while interpreting the results. First, the XVI system we evaluated has no bow‐tie filter, while OBI has full and half bow‐tie filters. From the fact that OBI unit is equipped with the bow‐tie filters and XVI is not (it is to be noted that the newest version of XVI is equipped with a bow‐tie filter), one may reasonably expect better image quality for the same patient dose on OBI, provided all other factors are comparable. However, measurements of half value layers for the X‐ray beams from the two systems led to the conclusion that the X‐ray beam generated by XVI is harder than that generated by OBI, even when the bow‐tie filters are used for OBI.^(1)^ Next, the quality of the image reconstruction, which in turn may depend on time taken by the reconstruction algorithm, will have an impact on the quality of the final image. XVI has different settings for quality of reconstruction. We used the medium resolution setting for all protocols for this study since this is the one that we use in our clinic. The high resolution reconstruction takes about 2 minutes longer to complete, which is the prohibiting factor for not using this mode in clinic. It is to be noted that the high resolution reconstruction setting will likely result in improved image quality characteristics at the cost of reconstruction time for the XVI images. Another factor that may affect image quality is the pixel resolution at which the images are reconstructed and imported. All the XVI images had 1×1 mm pixel resolution in the axial plane and 1 mm slice thickness. The half‐fan OBI images had 0.88×0.88 mm pixel resolution and full‐fan OBI images had 0.47×0.47 mm pixel resolution in the axial plane. All OBI images were acquired with 2.5 mm slice thickness. While there is some flexibility in selection of these parameters, our primary goal is to present image quality data by using typical clinical settings. It is also to be noted that there may be performance variations between different machines of the same model, and between different models for CBCT systems of the same manufacturers.

The first difference in our results is that the pixel values are stable when mAs is varied for OBI, while it is a function of mAs for all protocols for XVI. This indicates that while CT number calibration for OBI can potentially be performed by applying a constant additive correction (if at all necessary) that can be experimentally determined, such a relatively simple CT number calibration may not be possible for XVI due to the pixel value variation with mAs. It is possible that the quality of image registration will be enhanced for intensity‐based registration algorithms used for patient positioning, provided CT number calibration is performed for the CBCT. This is because voxels in the same region of the phantom will then have similar CT numbers in the planning CT and the CBCT. Additionally, CT number calibration could enable improved CBCT‐based dose computation for treatment dose, and dose‐adapted radiotherapy. This is a possible reason why CBCT based dose computation has already been investigated using OBI^(16)^ but not using XVI, to our knowledge, at the time of this study. Pixel value variation as a function of mAs in XVI is caused primarily by the detector response. The detector response is not linear as mAs increases, although one can calibrate the response to account for this. As previously mentioned, pixel values are calibrated to correspond to realistic CT numbers using CATPHAN phantom during installation for OBI. In the case of XVI, this is not done. Instead the imaging parameters are optimized (in terms of image quality) and set as clinical protocols and aren't flexible enough for end users to change (without affecting image quality).

From Fig. 6, it is seen that the OBI images are generally noisier. This is likely due to heavier postreconstruction filtering (such as average filtering) applied on XVI images. The average filtering is known to decrease noise but at the cost of decreasing low‐contrast resolution as well (since high frequency signal is erased). We don't know the filtering methods used by each system, but from the observation that OBI images generally have better low‐contrast resolution with noisier pixels, it seems highly likely that this is so.

From Fig. 8, it is observed that with all mAs settings for OBI half‐fan scans, S (Peripheral‐ROI) >S (Center‐ROI), so that there is a ‘cupping’ effect. The effect is more pronounced with the large phantom than with the small phantom. For the OBI full‐fan scans, S (Peripheral‐ROI) <S (Center‐ROI), so that there is a ‘capping’ effect. The ‘cupping’ effect occurs due to differential beam hardening through the central (harder) and peripheral locations of the phantom leading to higher beam intensity at the center of the detector than would be the case without beam hardening. For XVI, the effect is quite opposite. There is a ‘cupping’ effect with the smaller FOV scans (i.e., small phantom using head and neck protocol) and a ‘capping’ effect with the larger FOV scans (i.e., prostate, pelvis, and chest), except at low mAs. The ‘capping’ is markedly higher with the chest protocol (large FOV), exceeding 200 HU at all mAs. The high magnitude of ‘capping’ is attributed to software over‐correction for the cupping artifact when using the high mAs protocol.

For low‐contrast resolution test, the low‐contrast inserts were not visible on the XVI images obtained using medium resolution reconstruction quality setting used in the clinic, whereas their visibility was a function of increasing mAs for the OBI (full‐fan and half‐fan, both with the small phantom), under a similar mAs range examined. This observation may have some implication in terms of its utility in the performance of image‐guided RT where, in many instances, the target tumors to be visualized are of low‐contrast quality (e.g., ${\left(\frac{{\bar{\mu }}_{en}}{\rho }\right)}_{water}^{tumor}0.99$ ^(35)^).

It is difficult, however, at this time, to assess the clinical impact of this observation and the integrity of various IGRT processes, and would require further investigation before any conclusion can be drawn. The high‐contrast resolution appears to be approximately limited by the reconstructed image pixel resolution for the OBI, as well as for the XVI for the scan settings used in this study. Note that for the OBI half‐fan scan, the pixel resolution is 0.88 mm and for the XVI scans it is 1 mm, which respectively imposes bounds of ~ 6 and 5 lp/cm. Further improvement in high‐contrast resolution is observable using full‐fan scan on OBI only because of better pixel resolution of 0.47 mm. It is important to note that we did not use the high‐quality resolution setting for reconstruction of XVI images in this study due to their prohibitive reconstruction time for clinical use. The higher spatial resolution obtained using the high‐resolution setting provides scope for improved line pair visibility than that obtained by using the medium resolution setting (which was used in this work). The improvement would come at the cost of increased reconstruction time.

It is expected that a study of the image quality vs. mAs relationships presented in this work and the accompanying dose vs. mAs results in Song et al.^(1)^ can be used by physicists to develop good estimates of the dose‐to‐image‐quality tradeoffs in making responsible clinical decisions when establishing clinical scanning protocols for their respective needs.

## V. CONCLUSIONS

We have studied the impact of varying mAs on image quality for clinical protocols on two commonly used and commercially available CBCT systems. Various image quality parameters have been studied in order to assess image quality using extensive series of scans with image quality phantoms. As expected, most image quality parameters show improvement with the increase in mAs. The results presented in this work, together with the previously quantified dose‐mAs linear relationship for these systems,^(1)^ make possible estimations of the impact of dose on CBCT image quality for the two systems, and its subsequent optimization for use in IGRT.

## ACKNOWLEDGMENTS

This research has been supported by Elekta Oncology Systems.

## Supporting information

Supplementary Material Files
