# Supplementary figures and images for: An image quality comparison study between XVI and OBI CBCT systems
Source: J Appl Clin Med Phys. 2011 Feb 4;12(2):376–90. doi: 10.1120/jacmp.v12i2.3435 (PMC5718664; doi:10.1120/jacmp.v12i2.3435)

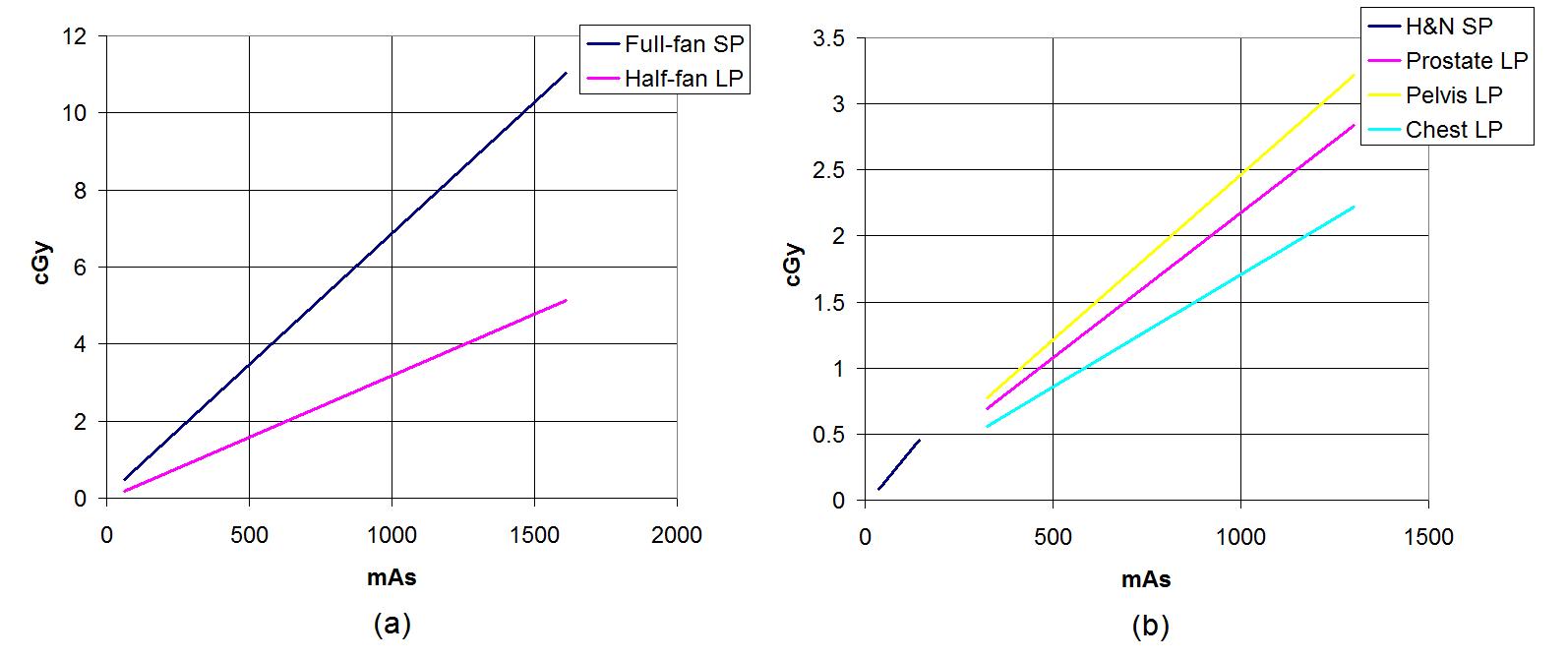

Supplement: Supplementary file 1 — Supplementary Material Files [file ACM2-12-376-s001.jpg]
